# Fast and accurate excited states predictions: machine learning and diabatization†

**DOI:** 10.1039/d3cp05685f

**Published:** 2024-01-09

**Authors:** Štěpán Sršeň, O. Anatole von Lilienfeld, Petr Slavíček

**Affiliations:** a Department of Physical Chemistry, University of Chemistry and Technology Technická 5 162 28 Prague Czech Republic stepan.srsen@vscht.cz petr.slavicek@vscht.cz; b Institute of Theoretical Chemistry, Faculty of Chemistry, University of Vienna Währinger Str. 17 1090 Wien Austria; c Vector Institute for Artificial Intelligence Toronto ON M5S 1M1 Canada; d Departments of Chemistry, Materials Science and Engineering, and Physics, University of Toronto St. George Campus Toronto ON Canada; e Machine Learning Group, Technische Universität Berlin and Institute for the Foundations of Learning and Data 10587 Berlin Germany

## Abstract

The efficiency of machine learning algorithms for electronically excited states is far behind ground-state applications. One of the underlying problems is the insufficient smoothness of the fitted potential energy surfaces and other properties in the vicinity of state crossings and conical intersections, which is a prerequisite for an efficient regression. Smooth surfaces can be obtained by switching to the diabatic basis. However, diabatization itself is still an outstanding problem. We overcome these limitations by solving both problems at once. We use a machine learning approach combining clustering and regression techniques to correct for the deficiencies of property-based diabatization which, in return, provides us with smooth surfaces that can be easily fitted. Our approach extends the applicability of property-based diabatization to multidimensional systems. We utilize the proposed diabatization scheme to achieve higher prediction accuracy for adiabatic states and we show its performance by reconstructing global potential energy surfaces of excited states of nitrosyl fluoride and formaldehyde. While the proposed methodology is independent of the specific property-based diabatization and regression algorithm, we show its performance for kernel ridge regression and a very simple diabatization based on transition multipoles. Compared to most other algorithms based on machine learning, our approach needs only a small amount of training data.

## Abstract 2

Efficient machine learning predictions for excited states can be achieved *via* machine-learned diabatization.

## Graphical abstract

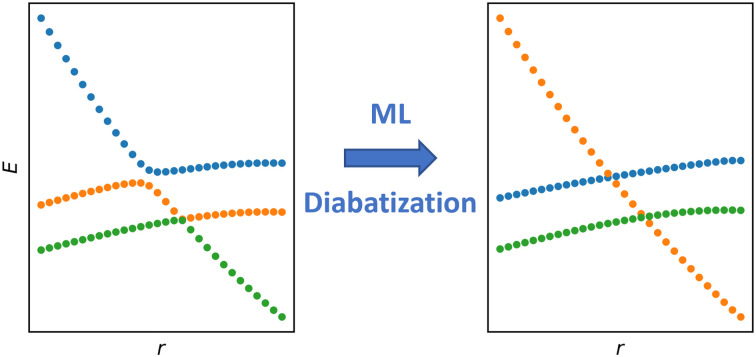

## 1 Introduction

Machine learning (ML) has been recently experiencing tremendous expansion in various fields of science and computational chemistry is not an exception.^1^ The motivation for using ML approaches is the high computational cost of quantum chemical calculations. We usually know how to obtain accurate results; however, such calculations are often computationally intractable and we have to settle with less accurate methods. ML can help us to shift the balance in favour of accuracy. Unfortunately, the applications of ML methods to electronically excited states have not yet reached the level of accuracy as the more common problem of dealing with ground-state properties.^2^ The fact that excited states are still an outstanding problem for ML is due to the high complexity of reference quantum calculations, high densities of states, and the fact that the predicted properties are not smooth in the vicinity of state crossings and conical intersections.^3^ We tackle here the problem of the low smoothness of excited-state properties.

Eigenfunctions and eigenvalues of the electronic Hamiltonian, which we usually get from electronic structure calculations, correspond to the so-called adiabatic representation. The states are ordered by their electronic energy for each nuclear configuration, resulting in non-crossing potential energy surfaces (PESs). While adiabatic states might become degenerate, they never truly cross if they have the same multiplicity. Electronic energies and other properties are then highly curved and non-differentiable. Low smoothness of the adiabatic basis represents a major problem for ML regression. Using a smooth diabatic basis, which allows for state crossings, seems like a natural solution how to improve ML efficiency. The two representations are connected through a geometry-dependent unitary transformation. Unfortunately, finding the diabatic basis is an outstanding problem itself. While the adiabatic basis can be obtained from a diabatic basis simply by diagonalization, the inverse procedure is highly complex as the diabatic basis is not uniquely defined. Even state-of-the-art methods such as fitting-while-diabatizing^4–6^ procedure usually require expert knowledge about the system and lots of manual work and expensive calculations. Dozens of various diabatization schemes based on nonadiabatic couplings (NACs) elimination, wavefunction smoothness, or properties smoothness have been proposed.^7–17^ To date, diabatization has been mostly limited to low-dimensional systems or specific wavefunction-based methods. However, several works attempting to solve the problem of automatic data-driven determination of the diabatic basis emerged during the last few years, predominantly based on neural networks.^18–22^ Very recently, an approach for the fitting of adiabatic energies of coupled surfaces avoiding diabatization by fitting coordinate-dependent coefficients of the characteristic polynomial of a potential matrix decomposition was suggested.^23^

While classical diabatization schemes are usually system-specific and very laborious, current ML-based approaches usually require lots of expensive training data and often a manual selection of reference geometries where adiabatic and diabatic bases coincide. We aim to combine the best of both worlds: we augment simple property-based diabatization schemes with an ML algorithm that corrects their deficiencies. As a result, we can obtain a smooth diabatic representation already with dozens or hundreds of samples. Note that our goal is not to compete with complex state-of-the-art diabatization schemes trained on huge samples in the accuracy of diabatic states. Instead, we utilize the proposed diabatization to improve the prediction accuracy in the adiabatic basis while using small training datasets. Therefore, smoothness is more important than for example exact locations of conical intersections, *etc.* Property-based diabatization is arguably the simplest category of diabatization techniques.^7^ It uses pairwise properties of adiabatic states such as transition dipole moments to obtain diabatic states whose characters change as slowly as possible. Unfortunately, there are some problems connected with this category of diabatization methods, which prevent their widespread application to larger molecules with multiple electronic states involved. First, we need to select such properties that allow the discrimination of all the involved electronic states. Second, ordering/labels of the states are not consistent throughout the configuration space as each nuclear geometry is diabatized separately: we get a set of diabatic energies and couplings (off-diagonal elements) for a given geometry and we have to assign them to the global diabatic states. The third issue arises from random signs of the electronic wavefunctions, which lead to random signs of the pairwise properties and further to random signs of the diabatic couplings. While the latter two issues can be easily resolved manually by inspection in one or two dimensions, it is impossible for a general multidimensional system.

The so-called cluster-growing algorithm has been previously proposed to correct the signs of diabatic couplings obtained with a different diabatization method.^24,25^ It uses a greedy ML-based approach and it gradually corrects the signs of neighbouring geometries, starting from a manually corrected initial cluster. While it proved to be useful for sign correction, it has not been used for the simultaneous correction of signs and state ordering, which is a significantly more complex problem. We identify two main problems connected with such applications: first, the manual correction of the initial cluster becomes cumbersome when dealing also with state permutations, especially for high-dimensional systems. Second, poor sign or state assignments can lead to a cascade of more wrong assignments as it is a greedy algorithm that makes only locally optimal choices at each stage. As we aim at smaller training samples, we can afford to overcome these limitations by employing a stochastic iterative procedure to reach convergence in our approach.

Within the proposed framework, we diabatize each geometry separately using property-based diabatization, and correct for inconsistent signs and ordering of diabatic states with the ML approach. The general idea of our approach is simple: properties in the diabatic basis should be smooth and smooth properties are easy to fit so we change the ordering and signs so that the properties are well-fitted with our ML model based on a combination of kernel ridge regression (KRR) and clustering. As a result, our methodology can extend the applicability of the whole category of property-based diabatization schemes to multidimensional systems with multiple states with as little as dozens of training samples. At the same time, we get an efficient way how to predict adiabatic energies, which can be obtained from the fitted diabatic states and couplings simply by diagonalization, and therefore save time on expensive *ab initio* calculations. While our ML algorithm can be in principle applied to any property-based diabatization, we propose here a series of simple diabatization methods based on transition multipole moments from the ground state as a byproduct. We also test the direct application of our ML algorithm without prior property-based diabatization, that is, testing whether ML prediction capabilities can be improved by simple reordering of adiabatic states. For example, recent research showed on the prediction of the energy gap between the highest occupied and the lowest unoccupied molecular orbital that prior classification can improve the smoothness of the fitted property and therefore ML performance.^26^

We focus here on the prediction of PESs but other properties can be predicted as well: atomic forces and approximate NACs can be directly obtained from the diabatic representation and other properties such as dipole moments can be fitted separately in the diabatic basis.^24,25^ We show the performance of the proposed methodology by reconstructing global PESs of excited states of nitrosyl fluoride and formaldehyde in thermally reachable regions at 300 K as we aim mainly at the application in modeling electronic spectroscopies. Using these small molecules for testing purposes allows us to use overlaps between all the states of all the sampled geometries for analysis, visualization, and benchmarking.

## 2 Computational methods

### 2.1 Property-based diabatization

We coupled our ML algorithm with property-based diabatization as it in principle the most straightforward approach to diabatization. Moreover, we propose here a series of very simple property-based diabatization methods which are easy to implement. Within the Born–Oppenheimer approximation, the eigenvectors of the electronic Hamiltonian are called electronically adiabatic states and the eigenvalues are called adiabatic PESs. The seam space of a conical intersection between two interacting states, formed by geometries with degenerate adiabatic energies, has *N*_int_ − 2 dimensions with *N*_int_ being the number of internal coordinates.^27^ It might seem that such a small subspace cannot play a significant role but the Born–Oppenheimer approximation breaks already for geometries in the vicinity of conical intersections and this is where radiationless transitions take place. When we aim to describe processes involving excited states, we usually have to go beyond the Born–Oppenheimer approximation. To do so, we have to calculate the probabilities of radiationless transitions usually expressed *via* NACs for states of the same spin multiplicity. However, these couplings are expensive to compute, often difficult to converge and exhibit singularities at conical intersection seams.

To get rid of the cuspidal ridges in PESs and other properties and singularities in NACs near conical intersections, we can switch to a different representation by applying a geometry-dependent unitary transformation matrix **T**(**R**):^28^1
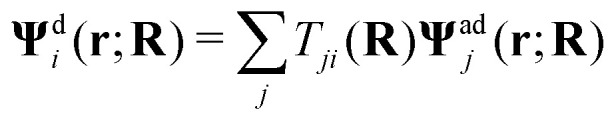

2 **U**(**R**) = **T**(**R**)^T^**V**(**R**)**T**(**R**)

where **Ψ**^ad^_*j*_(**r**;**R**) are the original adiabatic wavefunctions, **Ψ**^d^_*i*_(**r**;**R**) are the transformed diabatic wavefunctions, **V**(**R**) is the diagonal matrix of adiabatic PESs and **U**(**R**) is the transformed potential energy matrix (PEM) which is not diagonal anymore. The so-called strict diabatic basis would be obtained by such a transformation which would completely remove NACs. However, Mead and Truhlar^29^ showed in 1982 that the strictly diabatic electronic basis does not in general exist. Therefore, we have to settle with a basis that provides smooth elements of PEM and removes singularities in NACs. We call such a basis diabatic even though, strictly speaking, we should use the term pseudo-diabatic basis.

The diabatic basis is very convenient for ML applications as the diabatic PEM and also other properties are supposed to evolve smoothly with geometrical coordinates. At the same time, we can switch back to the adiabatic basis at any time simply by diagonalization. However, the non-existence of the strictly diabatic basis also means that the diabatic basis is not uniquely defined. Property-based diabatization schemes based on property unblending are the simplest and cheapest to apply. As diabatic wavefunctions are supposed to be smooth functions of geometry, we expect their properties to change smoothly as well. While enforcing global smoothness is a difficult problem, we can redefine the problem locally. Two crossing states become blended in the vicinity of a conical intersection and so do their properties. Property-unblending diabatization methods use this observation and make properties of the transformed diabatic states as different as possible which corresponds to the maximization of the following objective function:^7^3
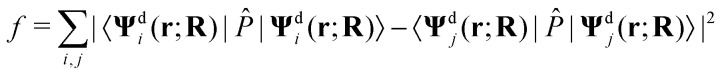
where *P̂* is the property operator. It has been shown that this maximization is equivalent to the maximization of the following objective function in the adiabatic basis:^30^4
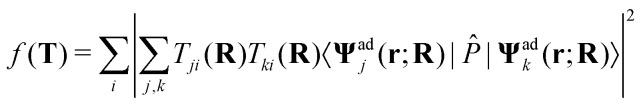
Many different property-unblending methods have been proposed using different properties to differentiate the states.^11,13,30–33^ It is important to note that the separation of matrix eigenvalues can be achieved by diagonalization.^7^ Therefore, the matrix formed by the eigenvectors of the property matrix corresponding to the *P̂* operator in the adiabatic basis can be used for diabatization. Unfortunately, this procedure leads to the above-mentioned problems with inconsistent ordering of the diabatic states and random signs of diabatic couplings.

The methodology proposed in this paper can be in principle connected with an arbitrary property-based diabatization method to extend its applicability to multidimensional problems. Nevertheless, we also propose here a series of simple and pragmatic property-based diabatization methods. The reasoning behind our methods is similar to the dipole-quadrupole^30^ (DQ) diabatization: we want to distinguish the electronic states based on their transition multipole moments. However, the DQ and similar methods require transition multipole moments between all pairs of states, which are not always easily available from electronic-structure calculations.^11,13,30–32^ For example, the popular TDDFT method based on the linear-response theory does not usually even yield the full matrix of (transition) dipole moments. One has to usually perform a separate calculation for each electronic state, which is both computationally demanding and laborious. It is even more problematic for higher multipole moments. We instead propose to form the property matrix based on inner products between transition multipoles from the ground electronic state, which are usually readily available.

This way, we form a series of methods, which we call transition dipole (tD), transition dipole and quadrupole (tDQ), and transition dipole, quadrupole and octupole (tDQO) diabatization depending on the highest multipole included. The property matrix **P** is then formed according to the following formulas, respectively:

5 *P*^tD^_*ab*_ = **μ**_0*a*_·**μ**_0*b*_

6 *P*^tDQ^_*ab*_ = **μ**_0*a*_·**μ**_0*b*_ + *ω*_Q_〈**Q**_0*a*_,**Q**_0*b*_〉_F_

7 *P*^tDQO^_*ab*_ = **μ**_0*a*_·**μ**_0*b*_ + *ω*_Q_〈**Q**_0*a*_,**Q**_0*b*_〉_F_ + *ω*_O_〈**O**_0*a*_,**O**_0*b*_〉_F_

where **μ**_0*a*_, **Q**_0*a*_ and **O**_0*a*_ are the transition dipole, transition quadrupole and transition octupole moments, respectively, as implemented in the PySCF^34,35^ code, version 2.0.1. 〈·,·〉_F_ is the Frobenius inner product, that is, the sum over the element-wise product. The weights *ω*_Q_ and *ω*_O_ can be set by hand or optimized within cross-validation or a similar procedure. However, for simplicity, we do not use here this flexibility and set all weights to 1. Also, we did not observe a significant improvement when tweaking the coefficients for our test molecules.

We do not claim these methods to be universal but they are pragmatic as they can be employed and tested very quickly. We can simply form the property matrix **P**, calculate the matrix of eigenvectors, and use it as the transformation matrix in eqn (2). The employment of these methods is reasonable as long as the ground is sufficiently separated from the other electronic states within the sampled configuration space.

### 2.2 ML-based reordering

Eventually, we want to correct the deficiencies of property-based diabatization but we start with a simpler problem: can we reorder the adiabatic energies for each geometry so that they form smoother surfaces than the original adiabatic PESs? We simply want to reorder the adiabatic electronic energies of each nuclear configuration so that they form new PESs that can cross where it is advantageous for learning. If the answer were positive, then we would be able to get better ML predictions of adiabatic energies without any underlying property-based diabatization. Also, such an algorithm can directly diabatize states of different symmetry since they cross without mixing, that is with zero NACs. Yet another motivation is the benchmark of our optimization procedure because we devised an alternative approach to solving this problem based on wavefunctions overlaps as described below, to which we can compare the results.

Direct optimization of the state ordering by the minimization of the prediction error is problematic as the variable state order introduces too much variability to the model, resulting in difficult optimization and overfitting problems. Overfitting might be reduced by introducing a regularization term penalizing the higher roughness/curvature of the predicted PESs. Nevertheless, we propose here a simpler clustering approach based on the expectation-maximization (EM) algorithm on which many common clustering algorithms such as *k*-means are based as well. By clustering, we refer here to the assignment of adiabatic energies of individual geometries to global states and their PESs. The main difference between our clustering and *k*-means is that we cluster the data by minimizing the prediction errors for each geometry instead of the distance to the centroid. Also, we impose the restriction that each adiabatic energy of a single geometry is assigned to a different global PES.

**Fig. 1:**
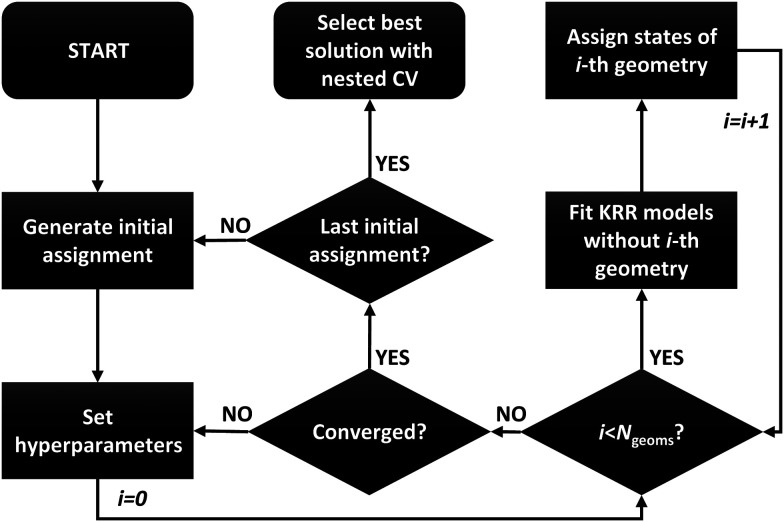
Flowchart of the proposed ML-based reordering algorithm.

A simplified flowchart of the optimization procedure is depicted in Fig. 1. We start the optimization from an initial ordering/clusters corresponding to some PESs, that is, either original energy-ordered adiabatic states or randomly shuffled states. The order of states for individual geometries can be seen as model parameters and we can use the EM algorithm to optimize them. We fix the state clusters and set KRR model hyperparameters in the expectation step and we use these fixed state clusters to estimate new ordering for each geometry separately in the maximization step. The excited states of each geometry are iteratively reassigned to the clusters in the maximization step by training a KRR model for each cluster corresponding to a single PES with the fixed ordering and hyperparameters but without the geometry which is currently being assigned. A distance matrix for the left-out geometry is then formed by calculating the prediction errors for its states using all the cluster KRR models. So we have a distance matrix between the energies of a single molecule and the state clusters and we want to find the best assignment so that the total prediction error is minimized. This is a common linear sum assignment problem, also known as the minimum (here maximum) weight matching. We solved this matching problem by the modified Jonker–Volgenant algorithm^36^ minimizing the mean squared error as implemented in the SciPy^37^ python package. We repeat this process of fixing the clusters, setting hyperparameters and estimating new state orders geometry by geometry until the clusters do not change anymore. Note that we observed better convergence by updating the clusters after the assignment of each geometry, a modification also applicable to *k*-means.^38^

Since the proposed clustering algorithm is stochastic and does not guarantee the global minimum, we start the optimization procedure many times from the original and also different randomly generated initial orderings. The number of initial conditions for the ML reordering optimization procedure was selected to obtain reasonably converged results and also to approximately match the results of the wavefunction-based reordering described below, that is, 1000 optimization runs. Since the results for different initial conditions are independent, the whole procedure can be efficiently parallelized. The obtained solutions are then compared by using cross-validation prediction errors and the best one is selected. However, the performance evaluated simply by the cross-validation prediction errors from KRR hyperparameters tuning (described below) is optimistically biased. The problem is when the same data are used to both select the model and tune the hyperparameters. We overcome this limitation by using nested (double) cross-validation, that is, the hyperparameters are optimized for each ordering in inner nested cross-validation. This way, we avoid the leakage of information from the training set to the test set.

Note that the proposed clustering algorithm is just one of the possibilities for how to perform the optimization. Alternatively, it is possible to optimize the ordering for instance by some metaheuristics such as simulated annealing or genetic algorithms. The advantage of the proposed ordering is its simplicity.

### 2.3 ML for property-based diabatization

The ML framework for state assignment outlined above is directly applicable to crossings between states of different symmetry which do not form conical intersections. Such states do not mix and the couplings are zero by definition. The seam has then the dimensionality of *N*_int_ − 1 with *N*_int_ being the number of internal coordinates and simple reordering of states is the optimal solution. The proposed algorithm, as defined in the previous section, can even improve the learning of states forming conical intersections with *N*_int_ − 2 dimensional seam as the algorithm can find a route through the conical intersections which provides smoother surfaces with more slowly changing characters of the involved states. In one dimension, for example, when following a trajectory or a scan, it simply decides whether it is advantageous for the learning to switch adiabatic states in the vicinity of the conical intersection depending on the number of training nuclear geometries. Nevertheless, the most efficient learning for conical intersections can be achieved in a diabatic basis.

We first apply a property-based diabatization yielding adiabatic PEMs with inconsistent state ordering and couplings' signs. We now want to modify the assignment step of the ML-based algorithm described above to obtain consistent order of states and signs based not only on diabatic PESs (diagonal elements) but also on diabatic couplings (off-diagonal elements). Mathematically speaking, for each iteration and nuclear geometry, we want to find such an assignment of its PEM **B** represented by a signed permutation matrix **S**, which minimizes the Frobenius norm to the predicted PEM **A** from ML models trained without that particular geometry:8
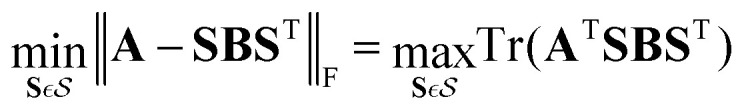
where ‖·‖_F_ is the Frobenius norm and 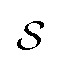 is the set of all signed permutation matrices. Unfortunately, this is not a linear sum assignment problem anymore because of the off-diagonal elements which couple the rows and columns together. This problem corresponds to the quadratic assignment problem (except the permutation matrices are signed) which is an NP-complete problem so there is no known algorithm for solving it in polynomial time. In fact, there are 2^*n*−1^*n*! signed permutational matrices for *n* states.

We can get an approximate solution by neglecting the arguably small diabatic couplings and using only the diagonal PESs; the problem then reduces to the linear assignment problem described in the previous section. However, we still need to correct the signs of diabatic couplings. The simplest approach is to compare all 2^*n*−1^ possible sign combinations for *n* states of each geometry and select the combination with the minimum error, an approach similar to phase-free learning of spin–orbit and nonadiabatic couplings by Westermayr *et al.*^39^ The assumption that the diabatic couplings are completely negligible compared to the diagonal terms is unnecessarily strict. We can use the result from such simplified optimization as a starting point for further optimization taking into account even the diabatic couplings. We use here an exhaustive search: we iteratively test all permutations of states and signs for every single nuclear configuration and choose the best-performing permutation with the smallest loss function. Note, that the search is exhaustive only in terms of states but it is iterative in terms of nuclear configurations. Also, the exhaustive search can be replaced by a 2-opt optimization if too many states were included.

Note again that different optimization procedures can be used. However, the main advantage of the iterative assignment on the leave-one-out basis is its simplicity and its reasonable resistance to overfitting.

### 2.4 Wavefunction-based reordering

To benchmark the ML algorithm and analyze the test cases, we propose yet another reordering algorithm based on wavefunctions, yet it is applicable only to direct reordering of adiabatic states and it cannot be used for the diabatic basis. The proposed wavefunction-based ordering is based on the assumption that the states preserve, at least to some extent, their character through the state crossings and conical intersections. As a result, wavefunction descriptors can be used to reorder the excited states of the sampled nuclear geometries in order to obtain states most preserving their characters. The most natural criterion for the similarity of electronic states is their overlap. Using wavefunction overlaps, we can define distances between all the electronic states of all the nuclear configurations representing the nuclear density.

As we have distances not only between nuclear configurations but also between the excited states, we can directly cluster the states. We propose here a clustering procedure based on the direct maximization of the silhouette coefficient. However, note that other clustering techniques can be applied as well; one has to only incorporate the condition that each state of a single nuclear configuration is assigned to a different cluster. The silhouette coefficient measures how similar are data points to other points within their own cluster compared to data points in other clusters. The silhouette coefficient can be calculated with any distance metric. In contrast, the most popular *k*-means algorithm cannot be used to cluster states based on overlaps as it requires the calculation of cluster centres.

We first define the distance of point *i* to its own cluster *C*_*I*_ and the closest different cluster, respectively:9
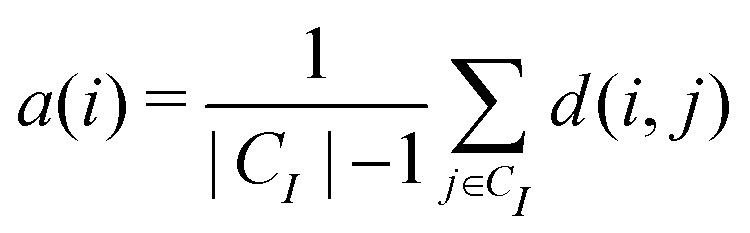
10
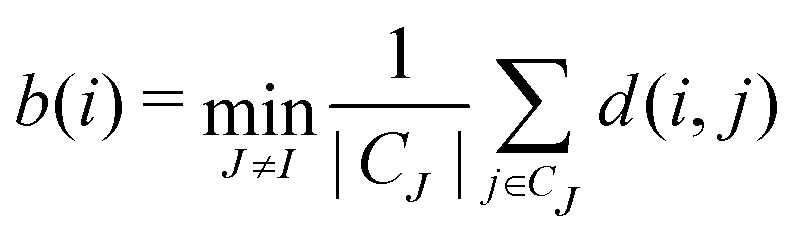
where *d*(*i*,*j*) is the distance between points *i* and *j* and |*C*_*I*_| is the size of the cluster *C*_*I*_. The silhouette for the given point is then given by these two quantities:11
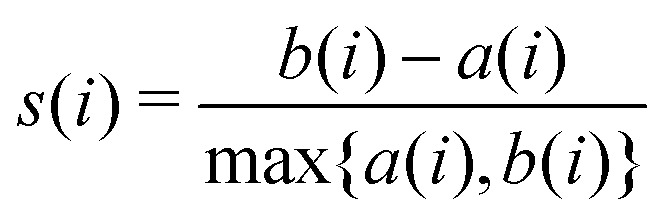
The mean silhouette over all states of all the sampled nuclear configurations represents our objective function to be maximized. Since the wavefunction overlap is a similarity metric, we define the distance by its complement to one:12
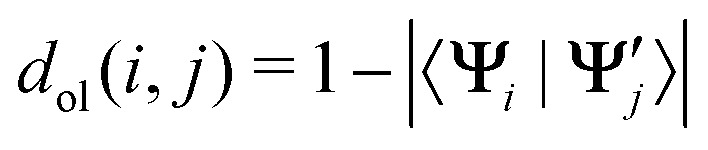
where **Ψ**_*i*_ and 
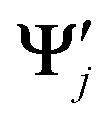
 are the wavefunctions of the two electronic states of two possibly different nuclear configurations. As wavefunctions can have arbitrary signs, we use the absolute value of the overlap. Alternatively, it is possible to use squared values or apply a phase correction.

We work here with CI-type wavefunctions which can be expressed as an expansion into Slater determinants:13
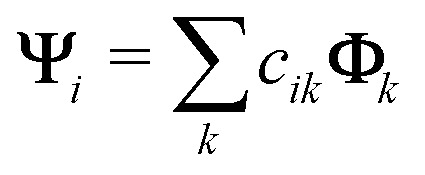
where *c*_*ik*_ are the CI expansion coefficients into Slater determinants **Φ**_*k*_. Note that this group of methods includes also popular time-dependent density functional theory (TDDFT), which can be written in the form of CI singles (CIS) expansion. The overlap is then given by the overlaps between the two sets of Slater determinants:14
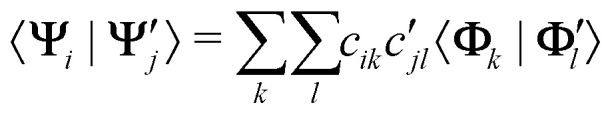
The overlap between two Slater determinants can be in turn expressed as a determinant containing overlaps between the constituting molecular orbitals (MOs):^40,41^15
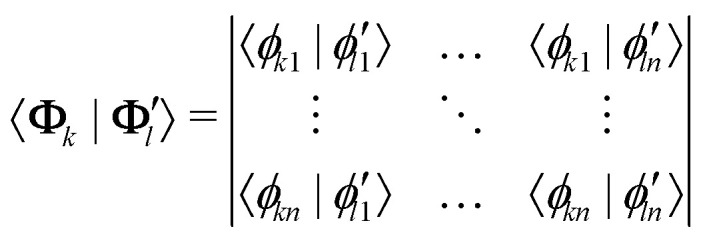


The calculation of wavefunction overlaps can be quite laborious and we need overlaps between all the states of all the geometries but this procedure serves here only to provide insight and validate the ML algorithm. Also, the geometries have to be aligned first in order to obtain meaningful values.

We start the optimization from the initial ordering/clusters, that is, the energy-ordered adiabatic states. Analogically to the ML reordering, we iteratively calculate the silhouette coefficient for each possible cluster assignment of each state separately for the selected geometry given the fixed clusters from the previous iteration. This way, we obtain a square matrix of silhouette coefficients between the states of the given geometry and the clusters and we select the best assignment again by solving the linear sum assignment problem. We iteratively repeat this procedure geometry by geometry until the clusters do not change anymore.

### 2.5 Regression model

Our ML model serves two purposes: we want to reconstruct diabatic PEMs and we want to predict adiabatic energies to reduce the number of expensive *ab initio* calculations. Many different regression models have been developed and their applications to excited-state simulations have been discussed.^2^ We make our models reasonably simple mainly for two different reasons: we want to keep our methodology clear and reproducible, and we need to perform the training many times during the correction procedure of the property-based diabatization so it has to be cheap. Therefore, we train a separate ML model for each adiabatic PES or each element of the diabatic PEM using the KRR method. KRR is a simple kernel method frequently used in quantum chemistry.^42,43^ Kernel methods use the so-called kernel trick that allows using linear regression algorithms to model nonlinear problems through an implicit transformation of the input data into a higher-dimensional space.^44^ In our case, the KRR method is the favourable choice because of its simplicity and efficiency for small training samples.

In KRR, the quantity of interest is predicted for feature vector **x** (molecular representation) using training samples **x**_*i*_ in the following way:^45^16
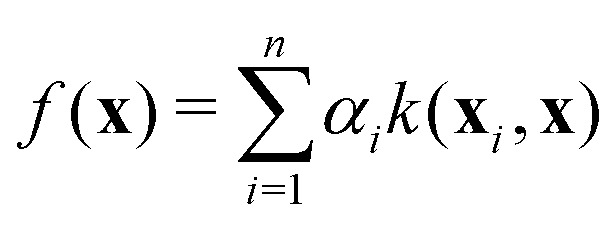
where *k*(**x**_*i*_,**x**) is a kernel function providing a similarity measure between the two vectors and *α*_*i*_ are the regression coefficients. We use here the Gaussian kernel which is especially popular in chemistry:^42^17
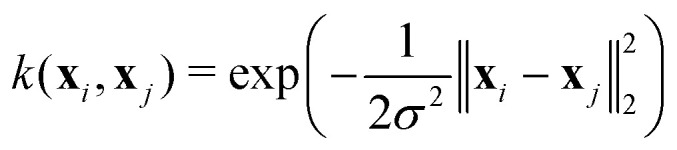
where ‖·‖_2_ is the Euclidean norm and *σ* is a model hyperparameter. The regression coefficients are obtained from the training data by the following minimization:^45^18
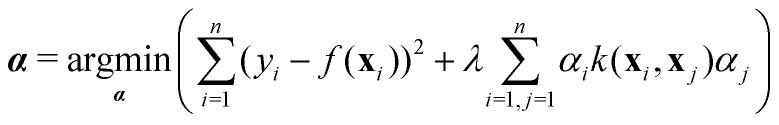
The first term is a common residual sum of squares. The second term including another hyperparameter *λ* is responsible for the regularization which should prevent overfitting of the training data. This minimization has a closed-form solution:

19 ***α*** = (**K** + *λ***I**)^−1^**y**

where **y** is the vector of known solutions for the training data and **K** is a kernel matrix with elements **K**_*ij*_ = *k*(**x**_*i*_,**x**_*j*_). This equation is in practice solved by the Cholesky decomposition. Within our approach, we need to solve this equation a lot of times, very often for the same or slightly modified kernel but with different **y**. This can be done efficiently by caching and/or updating the intermediate results of the Cholesky decomposition, effectively reducing the formal *O*(*n*^3^) scaling with the number of samples up to quadratic dependence. The hyperparameters *σ* and *λ* are selected on a grid using 10-fold cross-validation.

A crucial ingredient for the prediction of molecular properties is a molecular representation or molecular descriptors, that is, the feature vector **x** encoding the system, usually *via* the molecular structure.^43,46,47^ It should fulfil some basic requirements for ML to be efficient: it should usually possess translational, rotational, and permutational invariance.^48,49^ By working with nuclear configurations of a single molecular entity, some of the desired properties are automatically fulfilled. Namely, the number of atoms is constant, resulting in a constant-size molecular representation. However, as opposed to adiabatic properties, the diabatic PEM is not in general invariant with respect to permutations and inversion. Instead, it follows the symmetry of the corresponding complete nuclear permutation inversion (CNPI) group.^10,50,51^ While permutations do not play any role in the FNO molecule, formaldehyde belongs to the *C*_2v_(M) CNPI group, which is isomorphic to the *C*_2v_ point group.^52^ As a result, its diabatic states carry irreducible representations of the *C*_2v_(M) CNPI group. The symmetries of the involved system-specific irreducible representations can be directly incorporated into the fitted model.^5,52–54^ However, this can be a rather difficult task. We opted for a simpler option as we are interested mainly in more accurate predictions of adiabatic properties rather than accurate diabatic PEMs. By using a representation invariant to inversion and permutation of equivalent atoms, we effectively limit our model to a subspace of the whole configuration space. However, the rest of the configuration space is still given by corresponding CNPI symmetries. Moreover, the fitted subspace is sufficient if we are interested only in efficient prediction for adiabatic states as these are invariant with respect to both inversion and permutations.

We used a simple vector of normalized inverted internuclear distances as the molecular representation:^55,56^20
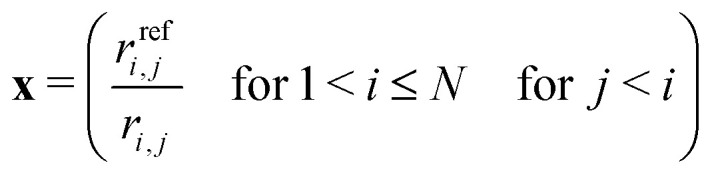
where *r*_*i*,*j*_ is the Euclidean distance between atoms *i* and *j* and *r*^ref^_*i*,*j*_ is the reference value. The reference values are usually taken from the minimal geometry but we used here average values sampled in the nuclear ensemble. This representation is simple yet efficient for our small molecules and it possesses both translational and rotational invariance. While it is also invariant with respect to inversion, it is not permutationally invariant. Same as Guan *et al.*,^57^ we enforce the permutational invariance for the formaldehyde molecule by permuting the hydrogen atoms so that the bond distances follow *r*_CH_1__ < *r*_CH_2__. Alternatively, we could use for example the permutationally invariant kernel.^58^

### 2.6 Computational details

The molecules were optimized at the B3LYP/6-31g* level with subsequent vibrational analysis on the same level using Gaussian G09,^59^ revision D.01. 1000 nuclear configurations for each molecule were subsequently sampled using the harmonic approximation and the temperature-dependent Wigner quasiprobability distribution:^60,61^21
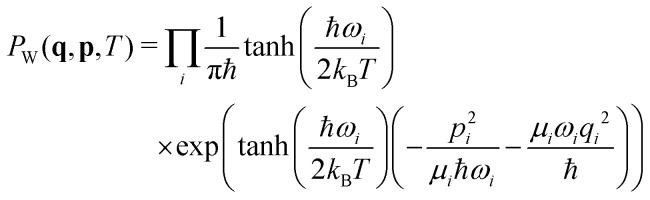
where *q*_*i*_ is the deviation along the *i*-th normal mode and *p*_*i*_, *ω*_*i*_ and *μ*_*i*_ are the corresponding momentum, angular frequency, and reduced mass, respectively. *T* is the temperature set to 300 K and *k*_B_ is the Boltzmann constant.

As described above, the hydrogen atoms were permuted for the formaldehyde molecule so that *r*_CH_1__ < *r*_CH_2__ to ensure permutational invariance. Moreover, we inverted the geometries so that the oxygen atom was always located on the same side of the CH_1_H_2_ plane for the calculation of overlaps to ensure invariance with respect to inversion. All the nuclear configurations for each molecule were geometrically aligned to one reference minimizing the mean square error between atomic centres *via* translation and rotation in order to obtain reasonable wavefunction overlaps needed for the analysis. Subsequently, the excited-state calculations for the sampled geometries were performed again at the B3LYP/6-31g* level of theory within the Tamm–Dancoff approximation in the PySCF^34,35^ code, version 2.0.1. Note that this level of theory does not provide quantitative results and the present calculations serve only to show the performance of the proposed algorithms. However, this level of theory combined with small test molecules allows us to calculate overlaps between all pairs of states of all sampled geometries, which is vital for the analysis and tuning of the optimization procedure.

## 3 Results and discussion

We chose nitrosyl fluoride (FNO) as the first example to show how the proposed methodology works. The first reason is that it is small so it can be easily analyzed but it is already a 3D problem that cannot be simply corrected by hand. The second reason is its *C*_s_ point group resulting in two sets of electronic states with either *A*′ or *A*′′ symmetry. We can therefore examine the behaviour of the algorithm both when two states of different symmetry cross without mixing and when states of the same symmetry form conical intersections. The second test case is the formaldehyde molecule which represents already a 6D problem but it is still possible to calculate pairwise wavefunction overlaps for analytical and benchmark purposes. Also, both molecules contain a set of singlet states that do not interact with other higher or lower-lying states at the employed level of theory, which is a prerequisite for efficient diabatization. States entering and leaving the predefined manifold represent a general problem for diabatization methods. Sampled geometries for both molecules, training indices, and calculated excitation energies and transition moments are included in ESI.†

### 3.1 Nitrosyl fluoride: 1D scan

**Fig. 2:**
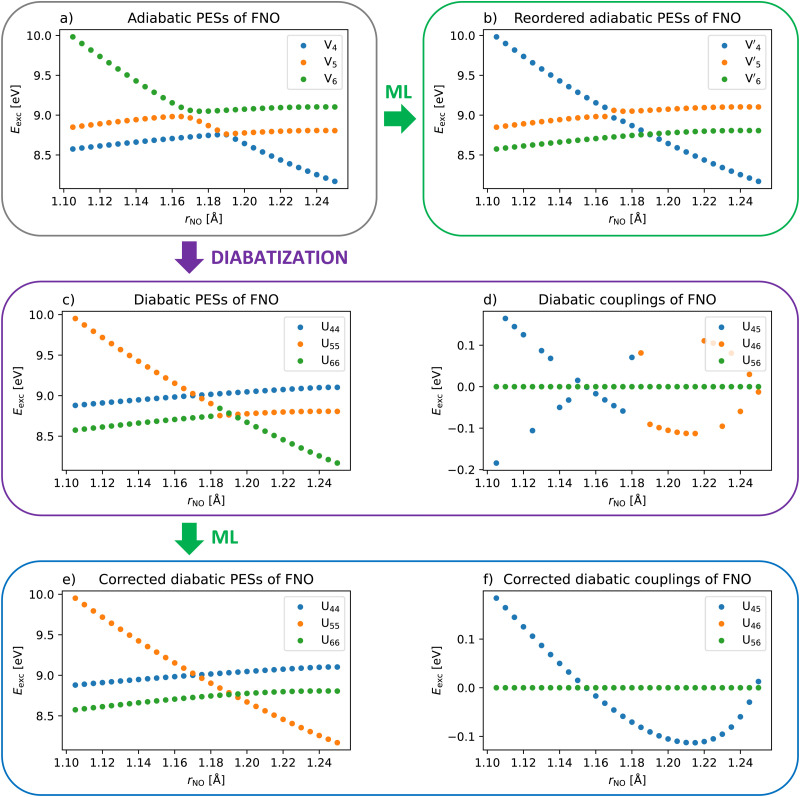
Excited states of the FNO molecule along the NO bond in the (a) adiabatic basis, (b) reordered adiabatic basis, (c) and (d) diabatic basis, and (e) and (f) reordered and sign-corrected diabatic basis.

Let us first look at the 1D scan of the FNO molecule along the NO bond to demonstrate how the proposed methodology works. The first three excited singlet states are all energetically well separated and do not mix or cross. We, therefore, focus on the next three states S_4_–S_6_ which cross and mix within the sampled configuration space. Note that these three states actually include the brightest states of the FNO molecule. We can see that while two states of the same *A*′′ symmetry form an avoided crossing, the third state has a different *A*′ symmetry a crosses them without any interaction (see Fig. 2a). We can directly apply the ML reordering algorithm without prior diabatization (see Fig. 2b). Such treatment correctly reconstructs the non-mixing diabatic state of different symmetry as the off-diagonal elements are zero and reordering actually represents the exact diabatization. The two states of the same symmetry switch their order in the centre of the avoided crossing resulting in two almost linear curves only with a small disruption located at the avoided crossing. While these states are not properly diabatic, they are much easier to fit than the original ones. Such a result looks encouraging; however, note that the 1D picture might be a bit misleading. The avoided crossing is caused by a conical intersection which cannot be displayed in one dimension. The reordering based on wavefunction overlaps is not plotted separately as it provides here the same result as the ML-based reordering but their agreement shows that the clustering works properly.

As a next step, we apply a simple tD diabatization scheme as outlined in Section 2.1. In this case, we need to distinguish only two states of the same symmetry along one coordinate so the property-unblending diabatization using just the transition dipole moments from the ground state is sufficient. Fig. 2c displays the diagonal elements of the diabatic PEM while Fig. 2d displays the off-diagonal elements, that is, the diabatic couplings. We can directly see the two problems of property-based diabatization: the ordering of the diabatic states is not consistent along the coordinate and the diabatic couplings have random signs. By the subsequent application of our algorithm, we get both smooth diabatic PESs and couplings (see Fig. 2e and f). One might point out that the correct ordering and signs are obvious. This is true in one dimension but the ordering and signs cannot be easily corrected by hand in a multidimensional space. Our algorithm allows applying property-based diabatization to multidimensional problems as shown below.

### 3.2 Nitrosyl fluoride: 3D case

**Fig. 3:**
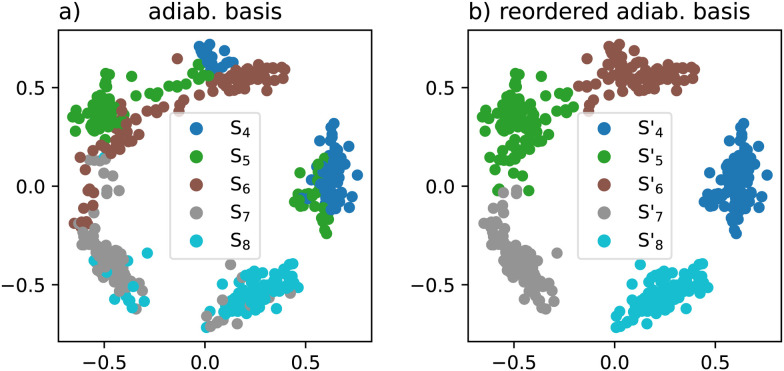
Multidimensional scaling projection of excited state clusters (a) before and (b) after reordering based on wavefunction overlaps for 100 nuclear configurations and 5 excited states. The projection corresponds to a 2D space in which the wavefunction overlaps are preserved as well as possible.

Let us now move to the full 3D space of the FNO molecule. In the full space, we have to include another two higher-lying states which interact with the three already included states. There are now two states of *A*′ symmetry and three states of *A*′′ symmetry. Fig. 3a presents a 2D multidimensional scaling projection of the five excited states for 100 nuclear configurations. Multidimensional scaling forms a low-dimensional representation of the data, in which the distances respect the distances in the original high-dimensional space as well as possible.^62^ We defined the distances the same way as in eqn (12) so the overlaps are also reasonably preserved given the limitations of a 2D plot. The excited states form five clusters corresponding to five diabatic states and none of them coincides with a single adiabatic state plotted with different colours. It can be clearly seen that the three states of *A*′′ symmetry mix together as there are samples connecting these clusters. On the contrary, the two *A*′ states do not mix suggesting that they are well separated within the sampled space.

To provide insight, let us first look at wavefunction-based clustering which serves here for visualization and benchmark purposes. Fig. 3b shows the same projection after we applied the wavefunction-based clustering described in Section 2.4. The adiabatic states of each geometry are now assigned to the clusters as well as possible. We can now create an ML model for each of these clusters instead of the original adiabatic states. The geometrical topologies of conical intersections are of course still present but we might hope that the new clusters present a better way through them. Nevertheless, these models serve mainly as a benchmark to test our ML reordering on an adiabatic basis before switching to a diabatic basis. Similarly, we reordered the adiabatic states using our ML approach to see whether such treatment is sufficient.

**Fig. 4:**
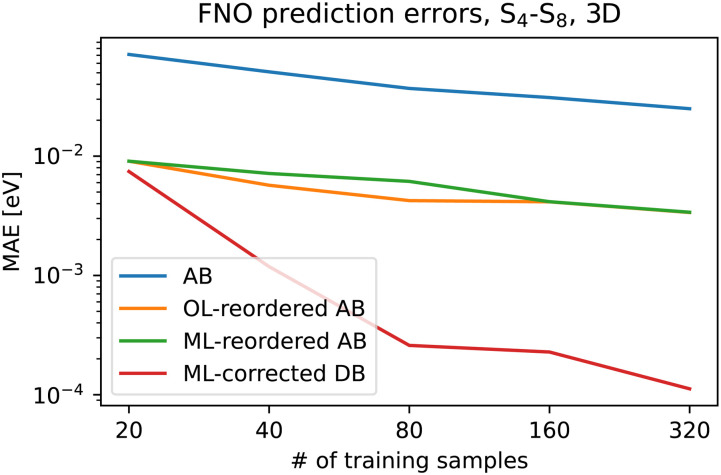
The mean absolute error of the kernel ridge regression for the FNO molecule as a function of training set size for adiabatic basis (AB), adiabatic basis reordered using wavefunction overlaps (OL), ML-reordered adiabatic basis, and values obtained from the diagonalization of ML-corrected diabatic basis (DB).

Finally, we applied the property-based diabatization and corrected the signs and ordering with our ML approach. The tD diabatization is not sufficient anymore as we need to differentiate 5 states. Therefore, we use here the tDQ diabatization. Let us now compare the accuracy of the ML prediction before and after applying all these methods, that is, original adiabatic states, adiabatic states reordered using wavefunction overlaps, ML-reordered adiabatic states, and ML-corrected diabatic states. The results are plotted for different training set sizes in Fig. 4. We always selected a training set of a given size, reordered/corrected it with the proposed algorithms, and used it to train a separate KRR model for each PES, and also each diabatic coupling in the case of the diabatic basis. We subsequently used these models to predict PESs for the rest of the 1000 geometries, which were not selected for the training set and evaluated the prediction error by means of the mean absolute error (MAE). In the case of the diabatic basis, the predicted PEMs are diagonalized and the resulting adiabatic energies are compared to the other models. Note, that the results are plotted on the log–log scale.

We can see that the improvement in accuracy is enormous for all the proposed approaches. Both adiabatic reordering approaches improve learning consistently almost by one order of magnitude. Also, both reordering approaches provide comparable results which suggest that our ML reordering procedure is sufficient. By switching to the diabatic basis and correcting the signs and ordering, we get another significant increase in accuracy. Not only that the absolute errors are much smaller but also the slope is better. The MAE is smaller by two orders of magnitude already with 80 samples.

**Fig. 5:**
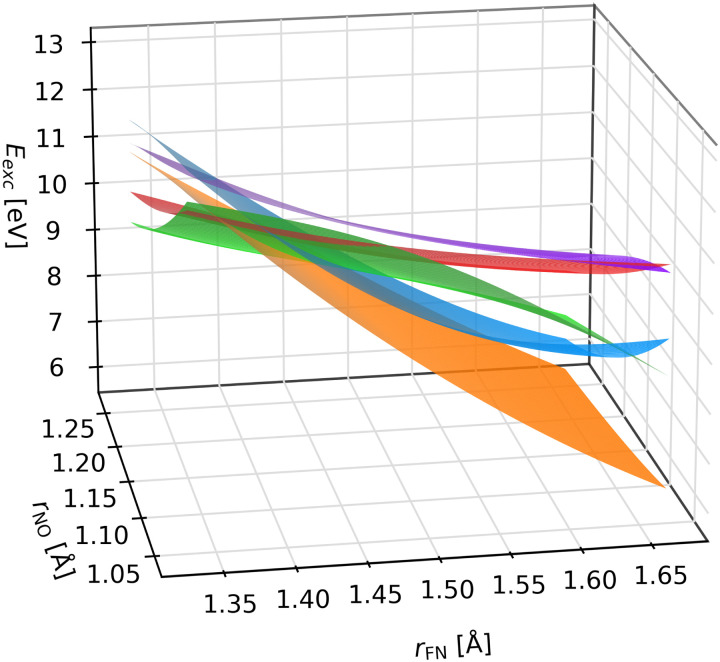
Potential energy surfaces obtained by the proposed ML approach based on the tDQ diabatization of the S_4_–S_8_ adiabatic states of the FNO molecule. The bond angle is fixed to 110°. ML models were trained on 320 geometries from the Wigner distribution.

To inspect how the diabatic states look like, we plot their PESs in Fig. 5 for a fixed bonding angle using ML models trained on 320 geometries. While it is difficult to plot five surfaces at once in a clear way, the PESs are clearly smooth and cross each other without forming conical intersections.

### 3.3 Formaldehyde: 6D case

**Fig. 6:**
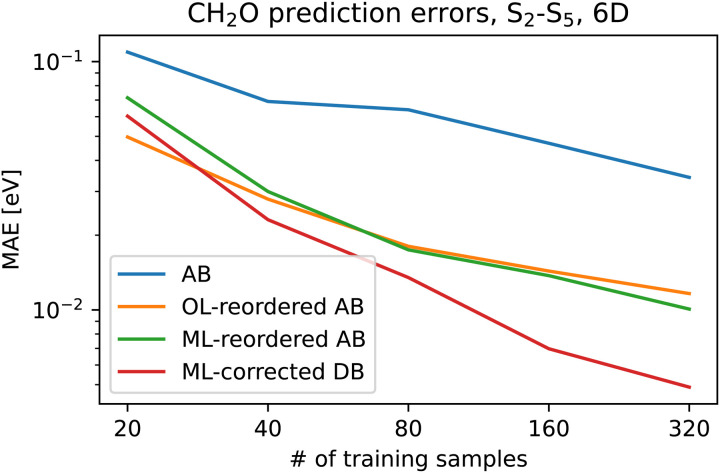
The mean absolute error of the kernel ridge regression for the formaldehyde molecule as a function of training set size for adiabatic basis (AB), adiabatic basis reordered using wavefunction overlaps (OL), ML-reordered adiabatic basis, and values obtained from the diagonalization of ML-corrected diabatic basis (DB).

We repeated the whole procedure for the formaldehyde molecule where we selected the tDQO property-based diabatization as the tDQ diabatization did not improve the learning. We could have in principle diabatized states of different symmetries separately for the FNO molecule but this is not the case for the formaldehyde molecule; while formaldehyde belongs to the *C*_2v_ point group in the minimal geometry, the symmetry is broken virtually for all the geometries. We included the S_2_–S_5_ states as those mix in the sampled region and are energetically well separated from both the S_1_ state and the higher-lying states at the employed level of theory. The MAEs for both adiabatic reordering approaches and the diabatic ML approach are presented in Fig. 6. We observe again a major improvement in prediction accuracy by up to one order of magnitude with 320 training geometries. While the improvement is not as remarkable as for the FNO molecule, one order of magnitude is still a huge improvement. It is important to realize that the final diabatic ML models are always limited by the underlying property-based diabatization. Both adiabatic reordering approaches decrease the prediction errors by up to half an order of magnitude and provide again very similar results.

## 4 Conclusions

We tackled two different problems at once: efficient machine learning for excited-state properties and diabatization. We proposed and tested methodology for correcting deficiencies of property-based diabatization techniques including random signs of the diabatic couplings and inconsistent ordering of the diabatic states throughout the configuration space, which prohibited the wider deployment of these methods to multidimensional systems. To this end, we developed a stochastic ML optimization procedure based on the combination of KRR and clustering. The optimization provided us with smooth diabatic states which are also easy to fit and predict. The set of adiabatic energies can be then easily obtained by diagonalization of the predicted diabatic PEMs. This way, we were able to improve the prediction accuracy by about 2 orders of magnitude in terms of MAE for the adiabatic energies of the FNO molecule and almost 1 order of magnitude for the formaldehyde molecule. We managed to efficiently utilize unprecedentedly small training sets including from dozens up to hundreds of nuclear geometries. However, it is important to note that the quality and performance of the final ML models are heavily dependent on the underlying property-based diabatization. Our ML approach corrects inconsistent state ordering and sings but it cannot correct for improperly chosen diabatization properties or state manifolds.

Our ML approach is applicable to any property-based diabatization. However, we also proposed a series of simple property-based diabatization schemes that are easily applicable even to single-reference methods such as TDDFT. These schemes are based only on transition multipoles from the ground state which makes them pragmatic and easily applicable but also not universal. The algorithm can be in principle applied to conical intersections of three or more adiabatic states occupying even lower-dimensional space whenever the underlying property-based diabatization is able to distinguish them. The direct application of our reordering algorithms without prior diabatization also improved the learning significantly: up to one order of magnitude for the FNO molecule and up to half an order of magnitude for the formaldehyde molecule. However, such behaviour cannot be probably expected for much more complex PESs of large systems.

Overall, we developed a methodology making diabatization more accessible for quantum-chemistry practitioners as it is based on the simplest category of diabatization methods, that is, property-based diabatization. The ML-corrected diabatic basis can save us many computationally expensive *ab initio* calculations as we can use much smaller training samples to achieve the same prediction accuracy. We also kept our optimization procedure as simple as possible for the sake of better transferability and reproducibility. Nevertheless, more efficient optimization procedures can be used for example by merging our algorithm with some metaheuristics; also, the cluster-growing^24^ algorithm can be used as the initial solution for the proposed optimization if we find a way how to form the initial cluster automatically. The methodology can be in principle used with different ML models instead of KRR. However, the ML model has to be reasonably efficient as it gets retrained many times during the optimization procedure.

This work opens the way to various applications. While we used the presented ML-corrected diabatization to fit PESs of two simple molecules, an analogous approach can be used to efficiently model electronic spectra using the nuclear ensemble method or any other property reflecting the ground-state geometry distribution.^25,63–65^ The proposed ML algorithm can be also directly used as an alternative to the cluster-growing algorithm to correct signs within other categories of diabatization methods as this particular problem is not specific only to property-based diabatization. Moreover, wrong state ordering was identified as a possible problem when learning differences between two electronic structure methods within Δ-ML.^3^ The basic reordering algorithm could resolve the issue caused by inconsistent ordering of adiabatic states at the two employed levels of theory. The present approach might be extended in the future to tackle also the problem with states entering and leaving the predefined excited-state manifold for diabatization by fitting a larger number of diabatic states (or predicting a smaller number of adiabatic states) than the number of input adiabatic states. Implicitly fitting a larger number of diabatic states within neural network architecture has been already shown to improve prediction accuracy.^22^ Eventually, the proposed diabatization might be in principle also used for efficient nonadiabatic dynamics simulations but one would have to take care that the configuration space is properly sampled and it might be advantageous to include gradients and NACs to the loss function if they are available for training. However, this application is yet to be explored. Also, such an application is in general more prone to the problem of states entering and leaving the predefined manifold.

## Data availability

Sampled geometries for both molecules, training indices, and calculated excitation energies and transition moments have been uploaded as part of the ESI.†

## Author contributions

Š. S.: conceptualization, data curation, formal analysis, investigation, methodology, software, validation, visualization, writing – original draft, writing – review and editing. O. A. V. L.: conceptualization, methodology, supervision, validation, writing – original draft, writing – review and editing. P. S.: conceptualization, funding acquisition, investigation, methodology, project administration, resources, supervision, writing – original draft, writing – review and editing.

## Conflicts of interest

There are no conflicts to declare.

## Supplementary Material

CP-026-D3CP05685F-s001
